# Psychological impact of far-right terrorism against Muslim minorities on national distress, community, and wellbeing

**DOI:** 10.1038/s41598-022-05678-x

**Published:** 2022-01-31

**Authors:** Kate G. Byrne, Kumar Yogeeswaran, Martin J. Dorahy, Jessica Gale, M. Usman Afzali, Joseph Bulbulia, Chris G. Sibley

**Affiliations:** 1 School of Psychology, Speech and Hearing, University of Canterbury, Christchurch, 8140 New Zealand; 2 Victoria University of Wellington, Wellington, New Zealand; 3 University of Auckland, Auckland, New Zealand

**Keywords:** Psychology, Human behaviour

## Abstract

The Christchurch mosque shootings on March 15th, 2019 was the deadliest incident of mass violence in New Zealand for over a century. The present study investigated the psychological impact of these terrorist attacks targeting a specific minority community on the psychological functioning of the wider New Zealand population by examining changes in terrorism anxiety, sense of community, psychological distress, and wellbeing. Data from the New Zealand Attitudes and Values Survey (*N* = 47,951; age range 18–99 years, *M* = 48.59, *SD* = 13.86; 62% female) collected across a year, including approximately 6 months following the terrorist attack, was used. Regression discontinuity analyses found a statistically significant increase in terrorism anxiety and sense of community following the attacks, yet counterintuitively, no significant change in psychological distress or wellbeing. These findings provide unique insight into the psychological implications of politically motivated violence for the wider population when terrorism is directed toward a specific minority group.

## Introduction

On March 15th, 2019, a far-right extremist in Christchurch, New Zealand, killed 51 Muslims and injured another 40 in two mosques. The perpetrator’s motivations, entrenched in White supremacy and eco-fascism, led him to target the Muslim community in their place of worship^1,2^. The attacks were especially notable given the relative lack of terrorism in New Zealand’s history over the past century^3,4^. Previous research suggests that terrorist attacks negatively impact psychological wellbeing, sense of community, and terrorism anxiety among the wider population^5–7^. However, such work has predominantly examined terrorist attacks perpetrated against the wider population, making it unclear if a terrorist attack targeting a specific minority community would similarly impact the wider population. Such terrorist attacks against minority communities, especially in their place of worship, have been a fairly common occurrence in recent years (e.g., 2018 Pittsburgh synagogue shootings, 2015 Charleston church shootings, 2012 Wisconsin Sikh temple shootings, 2019 Sri Lankan church bombings)^8^. To address this gap in understanding, we systematically investigate the impact of a terrorist attack by a far-right extremist against a minority Muslim community (representing ~ 1% of the national population) on the wider population’s psychological distress, well-being, sense of community, and terrorism anxiety following the 2019 Christchurch terror attacks.

### The effect of terrorism on the community

The United Nations^9^ defines terrorism as an act "intended to cause death or serious bodily injury to a civilian […] to intimidate a population, or to compel a government or an international organisation to do or to abstain from doing any act". Thus, terrorism is (a) characterised by the use of violence to promote behaviour change at the community or population level, and (b) weaponises trauma for gain. Research focusing on the psychological consequences of terrorism suggests that the aftermath of a terrorist attack is often characterised by confusion, fear, grief, and anger among the wider population, which can compound and contribute to general feelings of distress and anxiety^6,7,10–12^. Although it is expected that people with direct exposure to terrorism would experience increases in distress^13^, research suggests that people without direct exposure can also show changes in their psychological states^14^. For example, one study examining children residing 100 miles from the Oklahoma City bombing by an anti-government extremist revealed increases in post-traumatic stress disorder symptoms and functional impairment within 2 years of the attack through indirect exposure such as via the media^15^. Similarly, in a representative survey completed 11–13 days following the 2005 London bombings by Islamic extremists, 31% of Londoners reported substantial stress due to the bombings^11^. Furthermore, a cross-sectional study conducted one week after the 2016 Brussels bombings by ISIS extremists found 35.1% of Belgians reported moderate to severe anxiety and depressive symptoms^12^. Changes in mood and terror-related cognitions and behaviors were also found among the wider population 4 weeks after the 2015 ISIS terror attacks in Paris^16^. From a psychological perspective, contemporary terrorism can be particularly damaging because of the scope and speed of information sharing, including the possibility of livestreaming the violence like in the Christchurch attacks. This exposes masses of people to their own physical or existential vulnerability, undermining their sense of personal safety^10^. Put simply, terrorist attacks have a significant psychological impact on those not directly exposed to the attacks, both immediately afterwards, and sometimes months later^6,7,10–12,16–21^. However, it is presently unclear how terrorist attacks that target specific minority populations affect the psychological functioning of the wider population that is not targeted by the attacks. While a retrospective recall study 4–5 months after the 2011 Norway attacks in which a far-right extremist targeted members of a political party pointed to increases in the wider population’s negative emotions, these attacks were also directed at government buildings, and a community survey revealed that 1 in 4 people reported knowing someone directly impacted^21^. As such, the wider community had reason to identify with the targeted party leaving it unclear whether such effects would have arisen were a minority community targeted. If there were no risk of personal vulnerability at stake, would people have expressed negative psychological outcomes? The current research examines this question.

While terrorist attacks can adversely affect psychological functioning, there is also the potential for positive or adaptive change^22,23^. Of particular significance in the present context is how terrorist attacks influence a sense of social cohesion and solidarity within the population. For example, in the month following the 2004 Madrid train bombings by Al Qaeda, 85% of the city’s residents expressed feelings of solidarity with each other, with 82% reporting they felt included within a community^23^. Nonetheless, most research examines how a pre-existing sense of belonging can encourage resilience in the face of terrorist violence and not on how terrorist attacks subsequently affect one’s sense of community^24,25^. Here, we examine how terrorist attacks against a minority group impact the wider population’s sense of community.

### Importance of target group identity

The identity of the group targeted by terrorism may be significant in determining how sense of community, distress, and psychological well-being change in the face of terrorism. Indeed, although previous research provides a foundation for understanding the psychological impact of terrorism, how an attack by a member of the (arguably) dominant majority against a *minority group* affects the *general population* remains unclear. The September 11 attacks, the 2005 London bombings, or the 2015 Paris attacks, among others, were all directed towards the public at large with goals of inspiring terror among the wider population rather than a specific community. Nonetheless, the 2011 Norway attacks and the 2015 Charlie Hebdo shooting had more specific targets and research demonstrated elevated psychological distress at the population level as well as health impacts following these attacks^14,26–28^. However, there are differences between the targeted populations in these attacks and the population targeted in the Christchurch attacks, which may be significant for understanding the wider population level impact of terrorism. Specifically, the political party members and magazine staff targeted in the Norwegian and Charlie Hebdo attacks respectively may be perceived as representing mainstream institutions tied to the national identity of these countries making the attack seem like an attack on wider sections of the population. By contrast, a terrorist attack against a minority religious community may be seen in isolation, especially as it occurred in a place of worship for this specific group. It is important to note that the Charlie Hebdo shooting occurred within the broader Île-de-France attacks, one of which also included the Hypercacher siege that targeted Jewish supermarket patrons. Although Jews are also a religious minority like the Christchurch victims, as the siege occurred within a series of attacks, it is difficult to isolate the specific impact it had upon the French population, especially considering it occurred after the much-publicised Charlie Hebdo shootings. Thus, the present research provides valuable insight into the psychological and community impact of terrorism on the general population when the targeted group of the violence is a numerical and social minority group.

Therefore, the present study investigates the psychological impact the Christchurch mosque attacks had on the wider New Zealand population, using data from the New Zealand Attitudes and Values Study (NZAVS), an ongoing nationally representative longitudinal panel study. We specifically examined the impact of these attacks on terrorism anxiety, psychological distress, sense of community, and broader well-being to better understand both the general and specific implications of the attacks on the wider population. This data presents a broad perspective on the impact of terrorism across the general population when terrorism is specifically directed toward a small minority community. We use a regression discontinuity design (RDD) to analyse the outcome variables over an extended time period, approximately 6 months pre- and post-attacks. Such an approach allowed us to detect if there was any immediate or delayed change in the wider population’s psychological functioning following a terrorist attack against a minority group. This study, therefore, provides unique insight into how a terrorist attack targeting a minority community impacts on the wider population’s psychological functioning.

## Method

### Participants

Analyses were based on data collected by the NZAVS, an annual survey that has been tracking a nationally representative sample of adult New Zealanders since 2009. Participants were randomly selected through the New Zealand electoral roll which includes information for all adult citizens or permanent residents. Data reported here (*N* = 47,951) were collected between July 2018 and September 2019, encompassing the entire tenth wave of the NZAVS. Participants provided informed consent to take part in the study. Of the sample, 62% (*N* = 30,021) identified as female, 37% (*N* = 17,811) as male, and there was missing gender information for 0.25% (*N* = 119). Age of participants ranged from 18 – 99 years (*M* = 48.59, *SD* = 13.86). Ethnically, 88.62% (*N* = 42,495) identified as New Zealand European, 9.78% (*N* = 4,691) as Māori, 5.29% (*N* = 2,538) as Asian, 2.16% (*N* = 1,037) as Pacific Islander, and 3.79% (*N* = 1,815) as ‘Other’ (n.b., participants could identify with more than one ethnicity). Using the NZ qualifications framework, participants level of education was coded between 1 and 10 where 1 = no qualification and 10 = doctoral level qualifications (*M* = 5.33; *SD* = 2.73). A majority of participants had no religious affiliation (*N* = 28,624; 61.8%), and most participants reported being in a romantic relationship (*N* = 34,199; 71.3%). Of the sample, 6,944 (14.48%) were in the wider Canterbury region which includes the city of Christchurch, where the attacks took place. A more comprehensive description of the sampling procedure is presented in the technical document of the study^29^. The NZAVS was approved by The University of Auckland Human Participants Ethics Committee (Ref Number: 014889), and all research was carried out in accordance with relevant guidelines and regulations.

### Measures

#### Sense of community

 To measure sense of community, participants were asked to rate the item, “I feel a sense of community with others in my local neighbourhood” on a scale of 1 (“Strongly disagree”) to 7 (“Strongly agree”). The item was adapted from those used in the Quality of Life Survey^30^.

#### Terrorism anxiety

 Using a single-item measure from previous work^31^, participants indicated on a 7-point scale from Strongly Disagree (1) to Strongly Agree (7) the extent to which they concurred with the statement: “I often worry about terrorist attacks happening in New Zealand”.

#### Psychological distress

 Participants completed six items taken from the well-established Kessler-6^32^ measuring psychological distress (α = 0.86). On a scale of 0 (“None of the time”) to 4 (“All of the time”), participants indicated the extent to which over the preceding 30 days they felt ‘hopeless’, ‘depressed’, ‘restless or fidgety’, ‘worthless’, ‘nervous’, and felt ‘everything was an effort’.

#### Personal wellbeing

 To measure personal wellbeing, participants rated 4 items (α = 0.73) pertaining to health, standard of living, future security, and personal relationships from the Personal Wellbeing Index^33^ on a scale of 0 (“Extremely dissatisfied”) to 10 (“Extremely satisfied”).

The present study utilised a regression discontinuity design (RDD), which is used to test whether the application of a treatment at a given threshold of a continuous predictor associated with a continuous outcome yields a significant treatment effect. RDD is particularly useful in quasi-experimental designs such as the present study as it simultaneously tests for a treatment effect whilst also accounting for the overall trend in the continuous predictor and outcome^34^. RDD further accounts for a potential recovery function or return to baseline.

The treatment effect was tested by creating a time series wherein the day participants completed the questionnaire was used as a predictor for outcomes on the selected variables. The series was centred, with the day of the attacks (March 15th, 2019) represented as day 0 (i.e., the threshold). Thus, −1 represented March 14th, 2019, 1 represented March 16th, 2019, and so on. The discontinuity variable was represented using dummy coding wherein values of day < 0 were represented as *d* = 0 while values of day ≥ 0 were represented as *d* = 1. Thus, in the model, the value of *d* constitutes the difference between the predicted values of the outcome based upon the trend prior to the attacks and following the attacks. In order to clarify whether there were any immediate or delayed changes in the psychological functioning of the non-targeted wider community, we selected a wide time frame around the attacks to allow detection of any discontinuity that occurred during that time frame. A key assumption of the model is that those respondents who completed the questionnaire prior to the attacks were not systematically different to those who completed the questionnaire following the attacks (see supplementary materials for further details), allowing for any discontinuity to be attributed to the attacks as the most likely cause.

The model further incorporated 2nd, 3rd, and 4th order polynomials allowing for the modelling of potential non-linear trends both before the attacks and as a representation of a recovery phase (i.e., return to baseline). The post-attack trend was estimated by solving the interaction between the 1st to 4th order polynomials for day effect and values of *d* = 1. Within each model, the main parameter of interest is *d* as it constitutes the extent of any discontinuity in the series occurring at the time of the attack. Mplus, using Maximum Likelihood, was used to estimate the RDD model. The technical documents section of the NZAVS website provides full syntax for the model and equations^29^.

## Results

Table 1 presents the results for the separate regression discontinuity models predicting sense of community, terrorism anxiety, psychological distress, and wellbeing. A separate model was estimated for each outcome. Age, gender, ethnicity, education, religion, and region of residence were controlled in all models.

**Table 1:** Discontinuity Models of Each Variable.

Scale	*B*	*SE*	*β*	*T*	*P*
Sense of community	0.27	0.11	0.06	2.45	0.014*
Terrorism anxiety	0.59	0.12	0.12	5.13	0.001*
Psychological distress	− 0.01	0.05	− 0.00	− 0.18	0.859
Wellbeing	0.12	0.11	0.03	1.09	0.278

*Significant at *p* < 0.05.

There was a statistically significant increase in terrorism anxiety and sense of community following the attacks as shown in Table 1. For sense of community there was an increase of 0.27 units on the 1–7 scale immediately following the attacks, *p* = 0.014, 95% CI [0.05, 0.49]. For terrorism anxiety, there was a more marked difference with an increase of 0.59 units on a 1–7 scale immediately following the attacks, *p* = 0.001, 95% CI [0.35, 0.83]. In terms of effect size, these effects equate to values of 0.06 and 0.12 respectively (see Table 1). Although these effect sizes are small, they nonetheless represent a significant change following the attacks at the population level.

In contrast, the *d* parameters for psychological distress, and wellbeing were not statistically significant at the traditional *p* < 0.05 threshold, indicating no evidence for a statistically significant discontinuity in these parameters following the terror attacks (see Table 1; also see Supplementary Section for converging evidence using a distinct life satisfaction measure). These findings suggest that the terrorist attacks did not lead to significant change in the psychological distress and wellbeing among the non-targeted general population.

**Figure 1:**
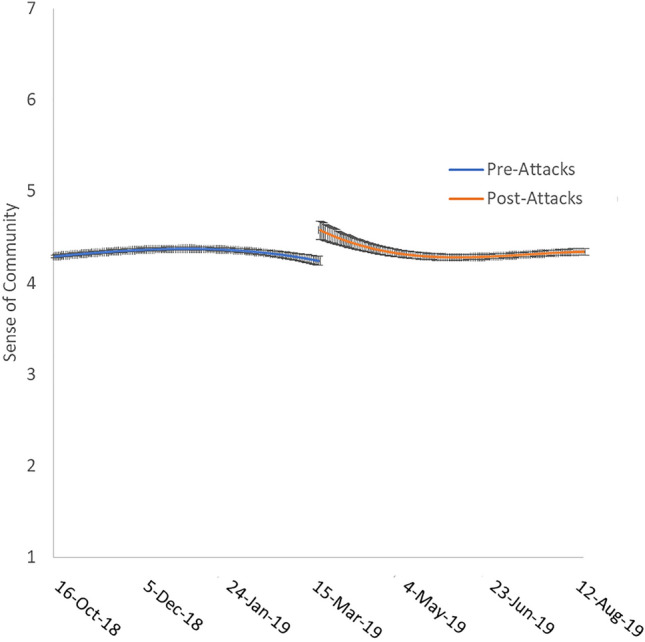
Sense of community approximately 150 days pre-attacks and 150 days post-attacks. Error bars represent standard error.

**Figure 2:**
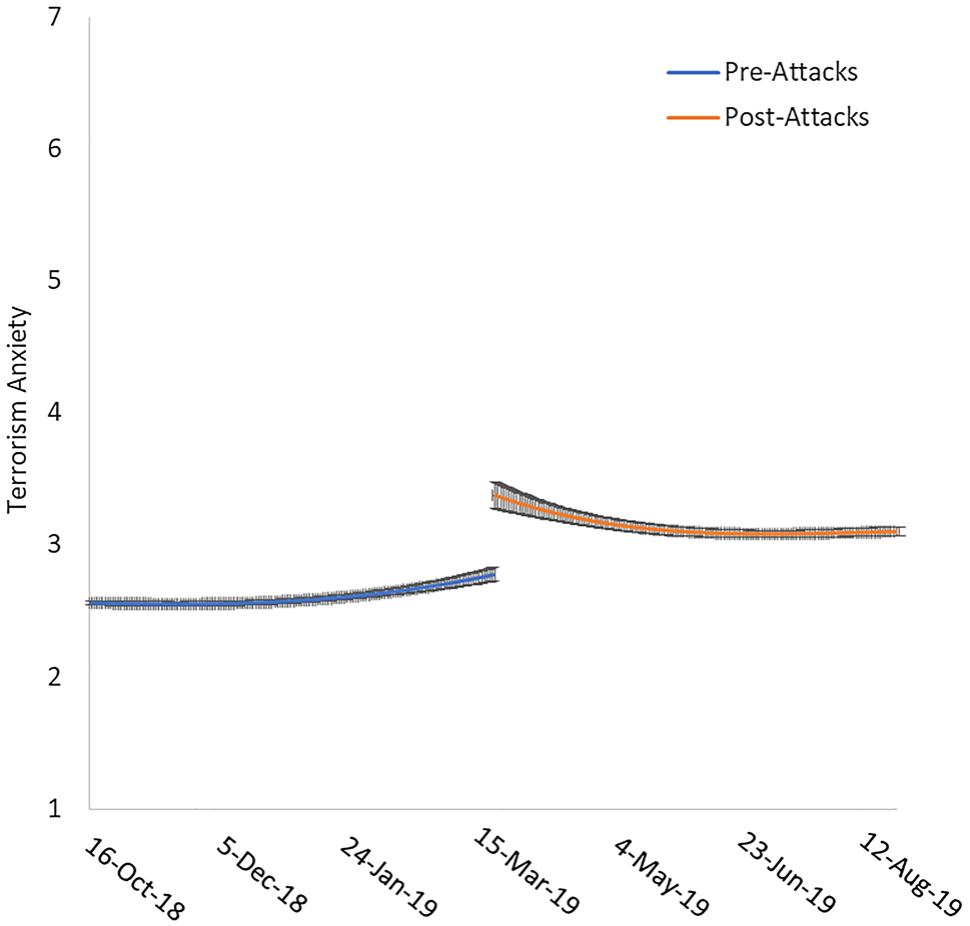
Terrorism anxiety approximately 150 days pre-attacks and 150 days post-attacks. Error bars represent standard error.

Figures 1 and 2 depict slopes for the rate of change across an approximate 300-day period centred on March 15th for the two statistically significant outcomes: sense of community and terrorism anxiety. As shown in Fig. 1, the slope for sense of community is relatively stable and flat prior to the attacks with a statitisically significant increase following the attack before gradually returning to baseline over approximately 60 days. Following the return to baseline, the slope remained flat and stable. This suggests that the terrorist attacks slightly increased sense of community as seen in previous research. However, such changes were short-lived and sense of community returned to pre-attack levels within less than 2 months.

As shown in Fig. 2, the slope for terrorism anxiety was initially flat with a slight upward trajectory before sharply increasing after the attacks followed by a gradual decrease over approximately 60 days to a new baseline higher than the initial pre-attack level. This suggests that the terrorist attacks not only lead to an immediate increase in terrorism anxiety, but may have established a slightly higher baseline level of anxiety that lasted months later.

**Figure 3:**
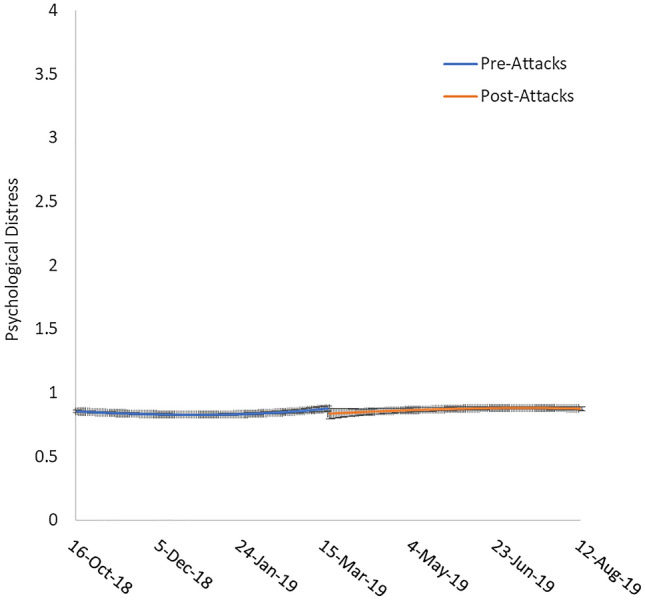
Psychological distress approximately 150 days pre-attacks and 150 days post-attacks. Error bars represent standard error.

**Figure 4:**
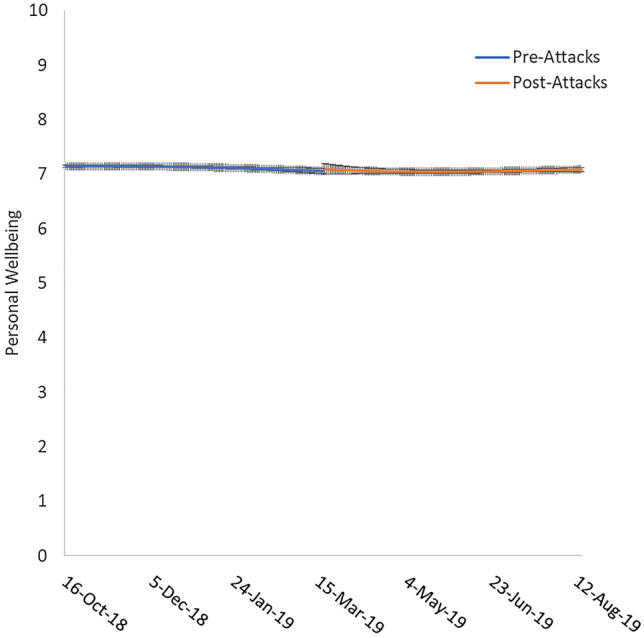
Personal wellbeing approximately 150 days pre-attacks and 150 days post-attacks. Error bars represent standard error.

Consistent with the results presented in Table 1, the slopes for psychological distress and personal wellbeing did not present a discernible discontinuity at the point of the attack (see Figs. 3, 4).

## Discussion

The current research systematically investigated the psychological impact of far-right terrorism against a minority community on nationwide terrorism anxiety, sense of community, psychological distress, and well-being. Previous research has found changes in the public’s emotions and cognitions following terrorist attacks. However, such work has predominantly examined the impact of terrorist attacks that were aimed at the general population^5–7,10–12,16–19,23,24,35,36^. The current work examines the psychological consequences for the wider community in the understudied context of White supremacist-driven terrorism targeting a specific minority community. Our study reveals that such an attack elicits a marked increase in both terrorism anxiety and sense of community following the attacks across the general population. However, we did not find evidence in this population for statistically significant changes in psychological distress or well-being. This finding contrasts with previous research examining the effects of other terrorist attacks, such as the 1995 Oklahoma City bombing, the September 11, 2001 attacks, 2005 London bombings, the 2004 Madrid bombings, the 2016 Brussels bombings, or the 2015 Paris attacks which found indicators of psychological functioning were worsened following a terrorist attack^6,15,16,19,35^.

The finding that terrorism anxiety increased following the mosque shootings is consistent with prior research showing that the aftermath of terrorist violence is associated with high levels of terrorism anxiety, often expressed as a fear of future terrorism^5,37^. As New Zealand had limited direct exposure to terrorism prior to the Mosque shootings, it is unlikely the population would have considered a terrorist attack imminent prior to the 2019 attacks^38^. This contextualises the increase in baseline terrorism anxiety following the attacks as it suggests the integration of this now understood vulnerability into the national psyche. Yet, the absence of change in psychological distress and wellbeing after the attacks, suggests this vulnerability did not impact more broadly on psychological functioning.

Importantly, it has previously been articulated that the impact of traumatic experiences on psychological functioning can follow several trajectories^39^. As data was gathered across nearly 6 months pre- and post-attacks, it allowed us to examine if there were any changes in the psychological functioning of the population both in the immediate aftermath and many months later. Additionally, our approach allowed us to detect multiple trajectories following the attacks. Although the findings may suggest resilience due to the seeming lack of significant distress, it is important to acknowledge that the use of the entire population potentially obscures useful findings relevant to specific trajectories that may exist within certain subgroups within the population.

The finding that sense of community was impacted by the attacks is consistent with previous work showing a sense of social cohesion and solidarity is often expressed in the wake of terrorist violence^23–25,40–42^. Notably, this finding is also consistent with some research conducted on social capital which has investigated the temporal development of social cohesion following terrorist violence^40,41^. It has been found that, while terrorist violence can foster social cohesion, where apparent, this increase is temporally limited, most evident immediately following the event, and eventually fades as the salience of the event declines^40,41^. An immediate increase in terrorism anxiety followed by a gradual return to baseline suggests that the psychological effects of terrorist attacks on the non-threatened general population tends to be short-lived. Our finding is significant given the relatively unique context of the Christchurch shooting where a specific minority group was targeted. This may suggest that terrorism can promote a sense of community regardless of what motivated the event and who was targeted. Further research into the drivers of this increase in social cohesion may elucidate whether the processes underlying the increase are the same across disparate contexts.

Conversely, the results for level of distress are inconsistent with prior findings that imply terrorist attacks impact community-wide psychological functioning both immediately after and even upto 6 months later^6,7,11,12,14,26,27^. The inconsistency between the current findings and previous work may reflect a difference in the context of the attack given in the Christchurch shootings the Muslim community was specifically targeted^6,7,11,12,16–19,35,43,44^. While some previous research has examined the implications of terrorism targeting a specific group, such as the 2011 Norway attacks and 2015 Charlie Hebdo shooting^14,26,27^, these were qualitatively different in that they involved attacks against mainstream institutions tied to the nation’s identity. This suggests that there are contextual factors surrounding the Christchurch shootings which produced a seemingly unique response in the general population. One potential explanation for our findings builds on a distinction between sympathy and empathy at times of tragedy. Sympathizing reflects acknowledging the suffering of others, where sadness and concern are expressed (i.e., feeling *for*) while empathizing reflects joining with their suffering, where similar emotions are felt at an affective level (i.e., feeling *as*)^36^. The public’s reaction to the terrorist attacks against the Muslim community was widely portrayed as an example for others to follow in the national and international media, as it involved an outpouring of support and warmth toward the community^45^. However, despite the population’s concern for the Muslim community as evidenced by acts of solidarity, measures of psychological distress and personal wellbeing did not increase. This suggests that non-Muslim participants, who constituted most of the sample, did not internalise the deeper emotions and responses felt by victims and the Muslim community more broadly. As Muslims represent a small portion of the national population (~ 1%), most of the nation’s population are unlikely to personally know a member of the targeted minority group. Moreover, the shootings involved an attack against the minority Muslim community within their place of worship. Thus, the general population may not experience the fear and anxiety evident in those who personally identified with the population targeted, as in previous attacks against the general population. Thus, sympathy rather than empathy might have underpinned the social cohesion and terrorism anxiety observed in the wake of the attacks.

Identificaton with the general population, but lack of identification with the targeted population, may help account for other findings, including the statistically significant result for terrorism anxiety. Concerns about terrorism were elevated after the attack, implying the general population was worried that a similar attack was possible; however, the lack of effect on psychological distress and personal well-being could suggest that the population may have been mostly concerned about a similar attack occurring again in New Zealand and not specifically toward themselves. Given the link observed between terrorism anxiety and mental health in previous studies^46^, it is possible that members of the non-targeted general population were not personally distressed as they did not fear being targeted. However, lacking measures of the specific groups people fear will be attacked, we can only speculate about the mechanism underlying this partial disparity of the results we report here in relation to previous studies. Future research is needed to clarify whether the distinction between empathy and sympathy may clarify the psychological responses we report here.

### Limitations and future research

The use of a nationally representative sample provides valuable insight into population level changes in psychological functioning around a significant event. However, limitations of the survey instrument constrain our power to examine the psychological mechanisms underpinning responses (such as sympathetic versus empathetic mechanisms). Another limitation of the current work is the use of the Kessler-6 measure for assessing psychological distress in combination with the timing of the assessments following the attack. The Kessler-6 asks participants how often they experienced distressing feelings over the preceding 30 days. With this wording, it is possible that distress may have been elevated immediately following the attacks, but in averaging over that time period, scores may have been reduced or inflated in relation to the event. However, given psychological research on peak experiences and trauma research suggesting that distressing events tend to become hypersalient^47^, participants would likely be more attuned to their feelings about the terrorist attacks if it had significant impact on them personally. Nonetheless, future research may benefit from the use of measures with more precise wording related to the specific attack.

Additionally, although RDD provides a valuable methodology to examine population level changes around a specific event, it requires a very high sample size to yield a sufficient degree of statistical precision^48^. Therefore, we are unable to examine the impact of the terrorist attacks on smaller communities within the national sample, including those with specific ties to the Muslim community. However, as the purpose of the current research was to examine population level impact of the attacks, future research would benefit from expanding this research to investigate the impact of terrorism on minority subpopulations.

Despite these varied limitations, the present study provides unique and important insight into the population-level psychological impacts of a terrorist attack upon a minority group. As such attacks are not uncommon, as evidenced in recent high profile events targeting minority communities (e.g., 2018 Pittsburgh synagogue shooting against Jews, 2015 Charleston church shootings against African Americans, 2012 Wisconsin temple shootings against Sikhs; 2019 Colombo church bombings against Christians), it is critically important to understand how such attacks impact the wider population when they target one specific community. Our findings raise questions about the general population’s capacity for perspective-taking and empathy following a terrorist attack against a minority group, even though they can express sympathy. This study would suggest that while genuine condolences and abhorrence may be expressed, and the general population may indeed be concerned about such an attack repeating itself, such concerns may not carry over to impact on the general population’s personal well-being or create prolonged distress. Future work should more systematically examine this distinction between the role of sympathy and empathy in the case of terrorism and mass violence directed at specific groups by distinguishing between short-term and longer-term changes in personal emotions and cognitions versus meta-perceptions about the target group. The current research provides a starting point for such future exploration on this topic. The present research also suggests that in the wake of terrorist attacks that target a minority group, mental health resources might be more efficiently channelled to support targeted groups rather than to the general population to maximize societal benefit.

## Supplementary Information

Supplementary Information.

## Data Availability

NZAVS data is hosted at the University of Auckland, New Zealand. Data cannot be made available due to ethical restrictions imposed by the University of Auckland Human Participants Ethics Committee. A de-identified dataset is available to appropriately qualified researchers upon request from the corresponding author, any member of the NZAVS advisory board, or the Chair of the University of Auckland Human Participants Ethics Committee.
