# Author Correction: Psychological impact of far-right terrorism against Muslim minorities on national distress, community, and wellbeing

**DOI:** 10.1038/s41598-022-06603-y

**Published:** 2022-02-08

**Authors:** Kate G. Byrne, Kumar Yogeeswaran, Martin J. Dorahy, Jessica Gale, M. Usman Afzali, Joseph Bulbulia, Chris G. Sibley

**Affiliations:** 1 School of Psychology, Speech and Hearing, University of Canterbury, Christchurch, 8140 New Zealand; 2 Victoria University of Wellington, Wellington, New Zealand; 3 University of Auckland, Auckland, New Zealand

Correction to: *Scientific Reports* 10.1038/s41598-022-05678-x, published online 31 January 2022

The original version of this Article contained errors. In the Measures section, under the subheading ‘Sense of community’,

“To measure sense of community, participants were asked to rate the item, “I feel a sense of community with others in my local neighbourhood” on a scale of 1 (“Strongly disagree”) to 5 (“Strongly agree”).”

now reads:

“To measure sense of community, participants were asked to rate the item, “I feel a sense of community with others in my local neighbourhood” on a scale of 1 (“Strongly disagree”) to 7 (“Strongly agree”).”

As a result of this error, the y-axis in Figure 1 is incorrect. Y-axis scale only runs from 1 to 7.

The original Figure 1 and accompanying legend appear below.

**Figure 1:**
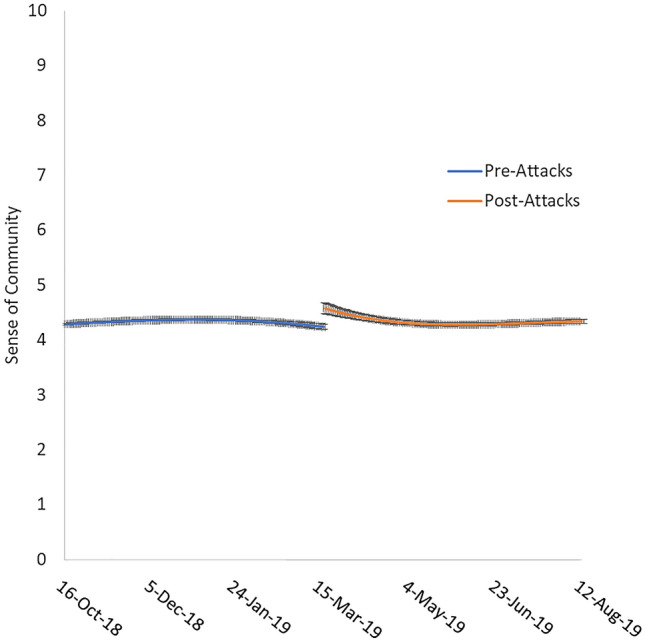
Sense of community approximately 150 days pre-attacks and 150 days post-attacks. Error bars represent standard error.

The original Article has been corrected.

