# Supplementary material for: Psychological impact of far-right terrorism against Muslim minorities on national distress, community, and wellbeing
Source: Sci Rep. 2022 Jan 31;12:1620. doi: 10.1038/s41598-022-05678-x (PMC8803852; doi:10.1038/s41598-022-05678-x)
Supplement: Supplementary file 1 — Supplementary Information. [file 41598_2022_5678_MOESM1_ESM.docx]

**Supplementary Materials**

**Life Satisfaction Analyses**

A 2-item measure of satisfaction with life adapted from previous research^1^ was also available in the data set. This measure was highly correlated with the subjective wellbeing measure reported in the main results (*r* = .69) and therefore these results are not presented as an independent measure in the main manuscript. However, in order to examine if there is converging evidence for our earlier findings, we used regression discontinuity design (RDD) with this measure. RDD analyses using this life satisfaction measure also reveals no statistically significant changes in life satisfaction, *B* = -0.02, *SE* = 0.08, *β* = -0.01, *t* = -0.20, *p* = 0.840. These findings occurred despite adjusting for all the same control variables as reported in the main results (i.e., gender, age, ethnicity, region, and religion).

**Multigroup Analyses**

**Table S1: Discontinuity models for each variable on subsample of White/European participants**

| Scale | *B* | *SE* | β | *t* | *p* |
| --- | --- | --- | --- | --- | --- |
| Sense of community | .32 | .12 | .07 | 2.77 | .006* |
| Terrorism anxiety | .64 | .12 | .14 | 5.50 | .001* |
| Psychological distress | -.02 | .05 | -.01 | -.38 | .702 |
| Wellbeing | .15 | .12 | .03 | 1.29 | .197 |

**Table S2: Discontinuity models for each variable on subsample of ethnic minorities (non-White/European participants)**

| Scale | *B* | *SE* | β | *t* | *p* |
| --- | --- | --- | --- | --- | --- |
| Sense of community | .48 | .40 | .09 | 1.22 | .222 |
| Terrorism anxiety | .48 | .47 | .08 | 1.04 | .299 |
| Psychological distress | -.16 | .18 | -.07 | -.92 | .357 |
| Wellbeing | -.07 | .41 | -.01 | -.17 | .866 |

**Table S3: Discontinuity models for each variable on subsample of participants with no religious affiliation**

| Scale | *B* | *SE* | β | *t* | *P* |
| --- | --- | --- | --- | --- | --- |
| Sense of community | .25 | .14 | .05 | 1.73 | .084 |
| Terrorism anxiety | .56 | .14 | .12 | 3.98 | .001* |
| Psychological distress | -.04 | .06 | -.02 | -.68 | .500 |
| Wellbeing | .23 | .14 | .05 | 1.61 | .107 |

**Table S4: Discontinuity models for each variable on subsample of participants with a religious affiliation**

| Scale | *B* | *SE* | β | *t* | *P* |
| --- | --- | --- | --- | --- | --- |
| Sense of community | .48 | .18 | .10 | 2.67 | .008* |
| Terrorism anxiety | .64 | .19 | .13 | 3.29 | .001* |
| Psychological distress | -.08 | .08 | -.04 | -1.06 | .288 |
| Wellbeing | -.07 | .19 | -.02 | -.40 | .693 |

**Demographic comparisons pre- and post- attacks**

**Table S5: No significant difference in religious composition of participants before and after the attacks: χ^2^(1) = 1.35, *p* = .245**

|  | Yes (*n*) | No (*n*) | Total (*n*) |
| --- | --- | --- | --- |
| Before | 14558 | 25622 | 40180 |
| After | 2349 | 4002 | 6351 |
| Total | 30021 | 17811 | 47832 |

**Table S6: No significant difference in gender composition of participants before and after the attacks, χ^2^(1) = 0.24, *p* = .626**

|  | Female (*n*) | Male (*n*) | Total (*n*) | |
| --- | --- | --- | --- | --- |
| Before | 26008 | 15458 | | 41466 |
| After | 4013 | 2353 | | 6366 |
| Total | 30021 | 17811 | | 47832 |

**Table S7: Statistically significant difference in ethnicity before and after the attacks, χ^2^(1) = 8.33, *p* = .004, such that there was a slightly greater percentage of participants reporting non-White ethnicity before the attacks (11.5%) relative to after the attacks (10.3%).**

|  | White/European (*n*) | Non-White/Euro (*n*) | Total (*n*) |
| --- | --- | --- | --- |
| Before | 36771 | 4798 | 41569 |
| After | 5724 | 658 | 6382 |
| Total | 42495 | 5456 | 47951 |

**Table S8: No significant difference in proportion of participants based in Christchurch versus the rest of New Zealand before and after the attacks, χ^2^(1) = 0.66, *p* = .417.**

|  | Christchurch (*n*) | Elsewhere (*n*) | Total (*n*) |
| --- | --- | --- | --- |
| Before | 3771 | 37798 | 41569 |
| After | 559 | 5823 | 6382 |
| Total | 4330 | 43621 | 47951 |

**Analyses revealed statistically significant differences in age before and after the attacks *t*(47947) = -14.08, *p* < .001, such that there was a small difference in average age among those sampled before the attacks (*M* = 48.24; *SD* = 13.91) relative to those after the attacks (*M* = 50.86; *SD* = 13.31).**

**References**

^1^ Diener, E., Emmons, R. A., Larsen, R. J., & Griffin, S. The Satisfaction with Life Scale. *Psych Assess,* ***49****,* 71-75 (1985).
